# Faible taux de succès du sevrage tabagique à court et moyen termes au décours d'un infarctus aigu du myocarde dans un service de cardiologie de Dakar au Sénégal

**DOI:** 10.4314/pamj.v10i0.72229

**Published:** 2011-10-10

**Authors:** Alassane Mbaye, Adja Mariétou Diop, Momar Dioum, Malick Bodian, Mouhamadou Bamba Ndiaye, Adama Kane, Nobila Valentin Yaméogo, Maboury Diao, Oumar Ba, Abdoul Kane

**Affiliations:** 1 Service de cardiologie, Hôpital Général de Grand Yoff, Dakar, Sénégal; 2 Clinique cardiologique, Hôpital Aristide Le Dantec, Dakar, Sénégal

**Keywords:** Tabagisme, sevrage, coronaropathie, risque cardio-vasculaire, Senegal

## Abstract

**Introduction:**

Le tabagisme est un puissant facteur de risque cardio-vasculaire. Son sevrage semble moins bien pris en compte chez les coronariens. Les objectifs de ce travail étaient d’évaluer la prévalence du tabagisme et le sevrage tabagique au décours d'un infarctus du myocarde dans un service de cardiologie au Sénégal.

**Méthodes:**

Il s'agit d'une étude transversale et descriptive réalisée entre janvier 2008 et juin 2010. Nous avons recruté les patients hospitalisés pour infarctus du myocarde puis les fumeurs actifs ont été inclus dans notre enquête. Les malades étaient sensibilisés pour l'arrêt du tabac puis suivis à 3 mois, 6 mois et 12 mois pour évaluer le sevrage tabagique.

**Résultats:**

Nous avons recensé 82 patients hospitalisés pour un infarctus du myocarde, parmi eux 26 sujets (25 hommes) étaient fumeurs (31,7%). L’âge moyen des sujets fumeurs était de 56±11,5 ans. La consommation moyenne de tabac était de 32±14 paquets-année et le score moyen de Fagerström de 8. Tous les patients ont arrêté le tabac pendant l'hospitalisation. Après un suivi de 3 mois, 45% des patients ont repris le tabac, 65% à 6 mois et 85% à 12 mois.

**Conclusion:**

Le tabagisme est assez fréquent chez les patients sénégalais présentant un infarctus du myocarde. Le taux de sevrage tabagique à court et moyen termes est faible. Le sevrage tabagique devrait alors constituer un objectif privilégié dans la prévention des maladies cardio-vasculaires.

## Introduction

Le tabac est un puissant facteur de risque de risque cardio-vasculaire. Sa prise en charge n'est pas toujours effective dans les suites d'un syndrome coronarien aigu (SCA) alors que le bénéfice du sevrage tabagique est important d'autant qu'il est plus précoce [1–4]. Dans les pays à économie faible, l'accent devrait être mis sur des méthodes de prévention moins onéreuses pour minimiser le coût de la prise en charge des maladies chroniques. Les objectifs de ce travail étaient d’évaluer la prévalence du tabagisme et son sevrage au décours d'un infarctus du myocarde dans un service de cardiologie de Dakar au Sénégal.

## Méthodes

Il s'agit d'une étude transversale, descriptive réalisée de Janvier 2008 à Juin 2010 au service de cardiologie de l'Hôpital Général de Grand de Dakar au Sénégal. Nous avons recruté les patients hospitalisés pour un infarctus aigu du myocarde puis les fumeurs ont été inclus dans cette enquête. Après consentement éclairé, les données furent recueillies sur un questionnaire complété par le test de Fagerstrom [5]. Les données relatives au tabagisme, les aspects sociodémographiques et cliniques ont été recueillis de même que les facteurs de risque cardio-vasculaire. L’évaluation du pic de monoxyde de carbone a été réalisée à l'aide d'un moniteur au laboratoire de physiologie de la faculté de Médecine de l'université Cheikh Anta Diop de Dakar. Les résultats exprimés en partie par million (ppm) permettaient d'apprécier grossièrement les délais de consommation du tabac ainsi que le degré de dépendance du patient. Au cours de l'hospitalisation, les patients ont été sensibilisés pour l'arrêt du tabac, ensuite ils ont été revus à 3 mois, 6 mois et 12 mois lors de la consultation de cardiologie et/ou de tabacologie ou par téléphone.

## Résultats

Nous avons recensé 82 patients consécutifs, hospitalisés pour un infarctus aigu du myocarde. Parmi eux, 26 sujets (25 hommes) étaient fumeurs (31,7%) avec un âge moyen de 56±11,5 ans. Les facteurs de risque cardio-vasculaire associés au tabagisme étaient dominés par l'hypercholestérolémie (51%), l'hypertension artérielle (11%), la sédentarité (15%), l'obésité (7%) et le diabète (4%). La cigarette était la forme de tabac la plus utilisée (92%) contrairement au tabac traditionnel (8%). La consommation moyenne de tabac était de 32±14 paquets-année et l’âge moyen de la première cigarette était de 18±5 ans [13–35]. Le premier contact avec le tabac a eu lieu au travail (62%), à l’école (19%) ou à la maison (19%). La dépendance à la nicotine était forte avec un score de Fagerström moyen=8. Le pic moyen de monoxyde de carbone était de 27±8 ppm. Les méthodes de sevrage tabagique par substitution ou par psychothérapie ont été utilisées respectivement chez 2 patients (7,7%). Un arrêt volontaire du tabac était observé chez tous les patients pendant l'hospitalisation. Au cours du suivi, 45% des fumeurs ont repris le tabac à 3 mois, 65% à 6 mois et 85% à 12 mois.

## Discussion

Nous avons réalisé une étude sur un nombre restreint de coronariens fumeurs, traités tous médicalement d'un infarctus du myocarde. La taille de notre échantillon ne permet pas des analyses statistiques puissantes, mais il nous permet d'apprécier la prévalence du tabagisme et d’évaluer nos pratiques de sevrage tabagique en particulier dans le post-infarctus du myocarde afin d'améliorer notre stratégie de prévention des maladies cardio-vasculaires. Nous procéderons dans la suite au commentaire de nos résultats.

Dans ce travail, nous apprécions la prévalence du tabagisme dans une population de coronariens sénégalais et avons analysé la prise en charge du sevrage tabagique et son suivi à court et moyen termes après un infarctus du myocarde. Le tabac constitue la plus importante cause de maladies graves évitables, parmi lesquelles les maladies cardio-vasculaires. La poursuite du tabac chez le coronarien est un élément de mauvais pronostic. En France, 25% des décès dus au tabac sont liés à une pathologie cardio-vasculaire, malgré cela 50% des coronariens restent fumeurs après un syndrome coronarien aigu [1]. En Afrique, le tabagisme constitue un facteur de risque fréquent d'infarctus du myocarde [6]. Au Sénégal, des études en population et en milieu hospitalier rapportent une prévalence du tabagisme située entre 16% et 36% [7,8]. Dans notre population de 82 patients hospitalisés pour un infarctus du myocarde, un tabagisme actif était noté dans 31,7% des cas avec une forte dépendance. Cette prévalence du tabagisme chez les coronariens est diversement rapportée dans la littérature [1,6]. Ce facteur de risque cardio-vasculaire est réputé plus fréquent dans l'infarctus du myocarde du sujet jeune [2,9]. S'agissant des études consacrées au sevrage tabagique après un infarctus du myocarde, elles s'accordent à souligner le bénéfice de l'arrêt du tabac tant sur la mortalité que sur la survenue d’événements cardio-vasculaires [9–12]. Cela souligne l'importance du sevrage tabagique dans la Le premier contact avecprévention secondaire de l'infarctus du myocarde. Sous l'effet de surprise de l'infarctus du myocarde et de la sensibilisation, tous nos patients ont arrêté le tabac dans les suites de leur hospitalisation. Cependant, le taux de récidive était important dès le troisième mois (45%) puis augmente au sixième mois (65%) et au douzième mois (85%) de suivi. Ce taux de sevrage est faible comparativement à ce que rapportent certaines séries notamment occidentales où les moyens utilisés pour le sevrage tabagique sont plus importants [13–16]. Plusieurs facteurs influençant le sevrage tabagique dans le post-infarctus ont été rapportés dans des séries différentes [13–18]. La forte dépendance nicotinique de nos patients combinée au faible taux d'utilisation des substituts nicotiniques et l'absence de programme d'aide au sevrage tabagique pourraient expliquer le faible taux de succès du sevrage tabagique. Cette dépendance physique et psycho-comportementale liée à la nicotine a été citée comme cause d’échec du sevrage tabagique ainsi que la survenue d'un syndrome anxio-dépressif au cours du syndrome coronarien aigu [3,13,15]. Vogiatzis et al [14] ont identifié plusieurs facteurs d’échec parmi lesquels l'absence de programme de sevrage, l'utilisation d'antidépresseurs, un antécédent de maladie vasculaire ou de bronchite chronique obstructive et un score de dépendance à la nicotine supérieur à 8 selon le test de Fagerström. Guevel et al [16] ont aussi rapporté que le stress était un facteur de reprise des habitues tabagiques chez 44,9% de leurs patients ayant récidivé. Dès lors et pour une meilleure efficacité, la sensibilisation à l'arrêt du tabac devrait commencer à l'hospitalisation du coronarien et devrait se poursuivre dans le cadre d'un programme intensif d'aide au sevrage tabagique [19]. La prescription de substituts nicotiniques pourrait alors aider à atteindre cet objectif [3,13,16]. Leur prescription précoce devrait être plus large comme le suggèrent certaines recommandations [13,16] qui préconisaient leur utilisation chez les coronariens fumeurs dès leur sortie de l'unité des soins intensifs. Cette suggestion contraste cependant avec la faible prescription, par les cardiologues français, de substituts nicotiniques [2,3]. Pourtant le rapport bénéfice/coût assez favorable pour le sevrage tabagique devrait faire du tabagisme une cible privilégiée de la prévention et à fortiori après un accident coronarien aigu [4,13].

## Conclusion

Le tabagisme est assez fréquent, avec une forte dépendance nicotinique, chez le coronarien sénégalais. L'hospitalisation pour infarctus du myocarde entraine l'arrêt immédiat du tabac mais le taux de sevrage à court et moyen termes est faible augmentant. Ainsi, le sevrage tabagique devrait constituer un objectif privilégié de la prévention des maladies cardio-vasculaires. Des méthodes de sevrage adaptées devraient être utilisées afin d'améliorer le sevrage du tabac chez ces patients déjà vulnérables.
